# Moved by Social Justice: The Role of Kama Muta in Collective Action Toward Racial Equality

**DOI:** 10.3389/fpsyg.2022.780615

**Published:** 2022-03-01

**Authors:** Diana M. Lizarazo Pereira, Thomas W. Schubert, Jenny Roth

**Affiliations:** ^1^Department of Psychology, Universitetet i Oslo, Oslo, Norway; ^2^Department of Psychology, University of Limerick, Limerick, Ireland; ^3^CIS-IUL, ISCTE-Instituto Universitário de Lisboa, Lisbon, Portugal

**Keywords:** kama muta, anger, sadness, collective action, racial equality, collective efficacy, social emotions, intergroup relations

## Abstract

Participation in collective action is known to be driven by two appraisals of a social situation: Beliefs that the situation is unfair (injustice appraisal) and beliefs that a group can change the situation (collective efficacy appraisal). Anger has been repeatedly found to mediate the relationship between injustice appraisals and collective action. Recent work suggests that the emotion of being moved mediates the relationship between efficacy appraisals and collective action. Building on this prior work, the present research applies kama muta theory to further investigate the relationship between efficacy appraisals and collective action. Kama muta is a positive emotion that is evoked by a sudden intensification of communal sharing, and largely overlaps with the English concept being moved. We investigated its relationship with collective action in both advantaged and disadvantaged racial groups in the context of the Black Lives Matter Movement (BLM) in Spring of 2020. In one pilot study (*N* = 78) and one main study (*N* = 215), we confirmed that anger toward the system of racial inequalities mediated between injustice and collective action intentions, and that kama muta toward the movement mediated between collective efficacy and collective action intentions. Both mediations were found for both Black and White participants. We also observed additional unpredicted paths from anger to kama muta and from efficacy to anger. Together, this provides evidence for the pivotal role of emotions in collective action intentions, but also points out that appraisals need to be better understood.

## Introduction

The Black Lives Matter movement (BLM) has become the largest social movement in the history of the United States (Buchanan and Patel, 2020). In 2020, the unfortunate events of racial discrimination against Black people had a massive impact worldwide. Black people and other racial-ethnic minorities have raised their voices to condemn the systematic racism that affects them on a daily basis. Meanwhile, many White people have stood up in solidarity in the fight for racial justice. This social movement is an example of how members of advantaged and disadvantaged groups can join efforts to act for social change.

Emotions play a pivotal role to motivate or undermining collective action efforts because they sit at the intersection of multiple determining processes: They influence decision-making and action (Montada and Schneider, 1989; Jasper, 2011). When shared, emotions can coordinate social interactions and cooperative behavior (Thomas et al., 2009b) and they may influence social identification, for instance by strengthening existing or promoting new group memberships (Peters and Kashima, 2007; Thomas et al., 2009a). This perspective of the role of emotions suggests that emotions experienced by both advantaged and disadvantaged group members could influence cooperation and foster intergroup equality (Thomas et al., 2009b) in contexts of collective action. Past work has described how several distinct emotions play a role in such processes (Fritsche et al., 2018). One emotion in particular has been focused on: anger. Research has studied anger (or resentment) as a mediator between injustice appraisals and participation in collective action (e.g., van Zomeren, 2019). Recent work in the context of environmental collective action has suggested that a second emotional experience, labeled being moved, can play a similar role, mediating between collective efficacy appraisals and collective movement participation (Landmann and Rohmann, 2020).

Building on these contributions, in the current research, we study intentions to participate in collective action toward racial equality of both Black and White US Americans. To follow up on the role of being moved in collective action, we use kama muta theory (Fiske, 2019; Zickfeld et al., 2019a). Kama muta theory theorizes the emotion underlying feelings of being moved based on social relations. We propose that this emotion can be experienced by both advantaged and disadvantaged groups and influence their intentions to participate in collective action. We thus extend general notions that positive social emotions can foster intergroup relations and promote social change by applying kama muta theory to collective action (Fiske et al., 2017; Pierre, 2019; Pizarro et al., 2021).

## Collective Action, Politicized Identities, and Social Movements

Collective action is defined as an action taken by a group of individuals to achieve a common goal (Wright et al., 1990b). Different behaviors such as participation in demonstrations, signing petitions or donating can be categorized as collective actions (van Zomeren and Iyer, 2009). They can also include unlawful non-violent or even violent actions.

Theoretical and empirical work has clarified that collective action often involves a change in social identification and the content of identity (van Zomeren et al., 2008, 2018). Politicized Collective Identity Theory (Simon and Klandermans, 2001) has been influential for this line of argument. Unlike broader group identities, politicized identities such as social movements include clear norms, beliefs and behaviors. Individuals acting on their basis understand themselves as acting on behalf of their group and with reference to a more inclusive societal context (Simon and Klandermans, 2001, p. 319). The theory suggests that an awareness of shared grievances (identifying illegitimate inequality and violation of principles as part of a group) and adversarial attributions (recognizing a common opponent to blame for the group predicament) are essential to acquire a politicized collective identity. Social movements such as the feminist movement or the BLM are examples of such politicized collective identities. It is important to clarify that politicized identity should not be confused with political identity or ideology (e.g., in the US American context, identification with Democrats or Republicans).

Collective action typically plays out among groups (here we use the terms advantaged and disadvantaged groups). Members of disadvantaged groups are usually more inclined than advantaged groups to challenge injustice through collective action (van Zomeren and Iyer, 2009). Consequently, most research has focused on how people directly affected by injustice take action to overcome it. However, some perspectives indicate that members of advantaged groups can also get involved in collective action in favor of the disadvantaged (Subašić et al., 2008; Craig et al., 2020). This can follow several processes. For instance, if members of advantaged groups focus on the other group and perceive their disadvantages as illegitimate, they can feel sympathy and reduce their ingroup favoritism (Harth et al., 2008). Another possibility is that members of advantaged groups start to identify with the disadvantaged group, for instance after perceiving violations of their moral convictions (van Zomeren et al., 2011).

Actions by members of advantaged groups may result from them adopting the aforementioned politicized identities as new social identities that cross the original group boundaries. As a result of shared beliefs and emotions, it is possible to create connections among people and to create new groups (Peters and Kashima, 2007). These shared cognitions are determinants of social identity (Swaab et al., 2007) influencing social interactions and action between groups (Tiedens et al., 2004). In sum, identification with a social movement that clearly defines shared beliefs, violation of principles, and a shared opponent provides a foundation for a common identification for different sub-groups and thus, could allow a shared emotional experience among them.

## Drivers of Collective Action

Collective actions depend on at least three different variables: identity, perceived unfairness, and perceived efficacy. Theorizing of such processes is grounded in classic theories of social identity and relative deprivation (Mummendey et al., 1999). According to the Social Identity Model of Collective Action (SIMCA), social identity predicts collective action directly and also indirectly through unfairness perceptions and efficacy beliefs (van Zomeren et al., 2008). Similarly, the Encapsulated Model of Collective Action (EMSICA) considers injustice and efficacy as predictors of collective action and points out social identification as a mediator in these associations (Thomas et al., 2009a).

A later model distinguishes two different paths for collective action and includes an emotional component explicitly. The dynamic dual-path model (van Zomeren et al., 2012) suggests that collective action is a strategy disadvantaged groups under certain circumstances use to cope with their disadvantage through two approaches. The first one is described as an emotion-focused path and places anger as a critical element. The second path relates to a problem-focused approach characterized by efficacy beliefs. Likewise, a recent extension of SIMCA (van Zomeren et al., 2018) considers the violation of moral beliefs and politicized identity as essential motivation to engage in collective action mediated through group-based anger and group efficacy beliefs, respectively. All these models have in common that they distinguish between an emotional path mediated by anger and a non-emotional path mediated by efficacy.

Recent contributions elaborate this approach by suggesting that emotions can be mediators in both paths. For instance, the Social Identity Model of Pro-Environmental Action (SIMPEA) acknowledges that various emotions may determine goals and actions (Fritsche et al., 2018). Further, some research proposes that similar to the mediating role of anger in the path of injustice to collective action, the relationship between collective efficacy and collective action may be mediated through emotions, suggesting hope (Wlodarczyk et al., 2017) and the feeling of being moved (Landmann and Rohmann, 2020) as mediators. We will expand on these recent contributions in the following sections.

### Appraisals as Predictors of Collective Action: Injustice and Collective Efficacy

Whether the aim is to change or defend the *status quo*, identity and perception of justice are important predictors of collective action (Thomas et al., 2020). Relative Deprivation Theory (RDT; Walker and Pettigrew, 1984) is widely used to explain the involvement of disadvantaged groups in collective action. RDT proposes that comparison with other groups and the subsequent perception of injustice could result in feelings of group deprivation that motivate collective action (Mummendey et al., 1999). New approaches extend the relevance of perception of injustice into a broader category of violation of moral beliefs (van Zomeren et al., 2018).

Advantaged groups would participate in supporting the disadvantaged group based on moral beliefs. For example, violation of a moral belief about justice would evoke emotional reactions such as moral outrage that motivate advantaged group members to act on behalf of the disadvantaged group (Radke et al., 2020). Since morality motivation could overlap with politicized identity (van Zomeren et al., 2018) it can lead to identification with the disadvantaged group (Craig et al., 2020) or with the social movement that represents those beliefs. Consequently, it can influence the collective action of both advantaged and disadvantaged groups toward the same goal.

Initially, efficacy was considered a subjective expectation regarding the effectiveness of the collective action (Klandermans, 1984). Subsequently, the role of the group in this process was widely acknowledged. Under the collective lens, group efficacy gives members of a group a sense of agency and empowerment necessary to believe that together they can change their reality (Drury and Reicher, 2005). Consequently, in disadvantaged groups, perception of instrumental social support increases the efficacy beliefs necessary to undertake actions to change their circumstances (van Zomeren et al., 2012). Similarly, for advantaged groups, perceived resources that include psychological, social, and political assets also predict their participation in collective action (Beaton and Deveau, 2005). Therefore, independently of the group’s status, collective efficacy beliefs are necessary to support a cause.

Furthermore, collective efficacy has a vital role in social identification. Some evidence suggests that collective efficacy could increase group identification by affirming and strengthening it (van Zomeren et al., 2010). If members of different groups come together for the same purpose, their beliefs about their group efficacy could increase and, thus, their sense of shared identity. The joint participation of advantaged and disadvantaged groups under the same cause could also influence hope that a change is possible. When hope is high, it can influence efficacy beliefs and motivate collective action (Cohen-Chen and van Zomeren, 2018). This approach suggests that participation of both advantaged and disadvantaged groups under the same cause would enhance collective efficacy beliefs and therefore, also reinforce a shared identity as part of the movement.

### Emotions: Linking Appraisals to Collective Action

Research has demonstrated the role of emotions in collective action (Montada and Schneider, 1989; Wright et al., 1990a; Mallett et al., 2008; Thomas et al., 2009b; Jasper, 2011; Tausch and Becker, 2013; Wlodarczyk et al., 2017; Cohen-Chen and van Zomeren, 2018). We focus on two emotions in the current research: Anger because it has been the one most consistently tied to collective action, and being moved because it has only recently been added as a potential predictor.

#### From Injustice and Norm Violation to Anger and Moral Outrage

Members of disadvantaged groups experience anger after perceiving injustice, which motivates collective action (van Zomeren et al., 2004, 2008). This perception of injustice elicits anger when the unfair situation targets oneself or others for whom one feels empathic concern (Batson et al., 2007). Therefore, advantaged groups can also experience this emotion on behalf of the disadvantaged (Mallett et al., 2008; Iyer and Ryan, 2009). Accordingly, identification with the disadvantaged group is associated with high reported levels of injustice and anger (Gordijn et al., 2006). However, anger can be experienced toward the advantaged group (Zhou and Wang, 2012). Alternatively, moral outrage blames a third party or system of inequality for moral transgressions (Montada and Schneider, 1989). As mentioned previously, blaming a common enemy is a key element of a politicized collective identity (Simon and Klandermans, 2001). Like anger, moral outrage is also provoked by the perception of injustice but is mainly elicited under violations of moral values such as justice and fairness, or other values derived from social relations (Rai and Fiske, 2011).

#### From Collective Efficacy to Being Moved and Kama Muta

Recent research investigated for the first time the role of being moved in collective action. Landmann and Rohmann (2020, p. 2) argued that “people may be moved and positively overwhelmed by the idea that they can collectively change something, and these feelings of being moved may, in turn, motivate collective action.” Thus, in their research, Landmann and Rohmann (2020) challenged the notion that anger (or the more general moral outrage) would be the only emotion involved in collective action. They suggest that experience collective efficacy causes being moved through a combination of moral, closeness, and achievement appraisals, referencing various theoretical perspectives of being moved (for an overview of theories on being moved, see Zickfeld et al. (2019b)). Consider a typical measurement item for collective efficacy: “I think that together we can change [the group-related problem].” While previous conceptualizations put the focus on “can change,” Landmann and Rohmann’s proposal emphasizes the “together” part and the emotional impact experiencing togetherness can have.

Landmann and Rohmann (2020) found support for their model in two studies. The first study investigated members and sympathizers of a specific local but widely known ecological movement in Germany; the outgroup for this group was a coal mining company. The second study investigated psychology students from a German university regarding views of the same movement. The second study also manipulated injustice and efficacy appraisal and focused on authorities as the outgroup. The results are comprehensive because all variables were measured both on a situation-specific and a general level, and there is both correlational and experimental evidence. As predicted by the modified model, feelings of being moved influenced collective action behavior *via* the path of collective efficacy. This path works in parallel to the path from injustice appraisals to anger to collective action. In addition, there was some crossover between the paths: feelings of being moved influenced collective action also *via* injustice appraisals. Being moved was measured with the German terms “bewegt” (“moved”), “überwältigt” (“overwhelmed”), and “ergriffen” (“stirred”). Being moved was distinguishable from sadness, which was measured separately.

Being moved has received increased attention in recent years. The literature is not without its controversies (Zickfeld et al., 2019b). Some authors have considered being moved as mixed emotion that incorporates both positive and negative affect, or that comes in two variants, one associated with positive affect and one associated with negative affect (Bartsch et al., 2014; Menninghaus et al., 2015; Landmann and Rohmann, 2020). Others have argued that it is a positive experience that can or cannot co-occur simultaneously with negative feelings (Menninghaus et al., 2015). The exact appraisal is debated as well. Proposals vary from broader notions such as surpassed internal standards in either relationships, achievement, willpower (Landmann et al., 2019), fit with core positive values (Cova and Deonna, 2014), and compatibility with prosocial norms and self-ideals (Menninghaus et al., 2015) to more circumscribed ideas such as salience of affiliative attachments (Cullhed, 2020).

Some of this debate may arise because the English vernacular term “being moved” can be used both for a very specific emotional experience, but also for a broader category of emotions with fuzzy boundaries, even including emotions like sadness (Zickfeld et al., 2019b). As an alternative to studying this emotional phenomenon, kama muta theory proposes to start with a theoretical definition rather than with emotion labels, following a tradition in social psychology of introducing separate terms for theoretically defined emotions (e.g., dissonance and elevation). Kama muta theory conceptualizes the experience denoted by the more narrow usage of “being moved” under the artificial (originally Sanskrit) term *kama muta* (*being moved by love*) (Fiske et al., 2017, 2019). It proposes that kama muta is elicited by appraisals of sudden increases in communal sharing relations (i.e., social closeness around perceived shared essence; Fiske, 1992); that it is positive; that it goes along with physical manifestations such as weeping and goosebumps when intense; and that it can lead to a sense of collective identification and motivate affective devotion and moral commitment. These hypotheses have been supported by cross-cultural studies (Zickfeld et al., 2019a).

Note that there are a few differences between kama muta theory and how Landmann and Rohmann use their concept *being moved*. Kama muta theory proposes intensifications of communal sharing as the primary appraisal, and argues that appraisals of exceeded morality or achievement should only lead to kama muta if they themselves affirm a communal sharing relation. For instance, athletes might be crying after victory because they see the victory as an affirmation of the communal relation to their family that sacrificed a lot for enabling them to train, or as an affirmation of the inclusion of the disadvantaged group they are a member of. However, this claim of kama muta theory has never been thoroughly tested. Also, neither Landmann and Rohmann (2020) nor the current work actually include specialized measures of these mediating variables. These differentiations between the theoretical approaches thus remain theoretical for the current work.

We nevertheless prefer to use the concept of kama muta over the term being moved in the current paper for two reasons: (1) We want to avoid using a vernacular concept from one specific language that may or may not be the language that is actually used in the measure (Fiske, 2020). (2) We want to emphasize that we are not referring to the broad meaning of the English vernacular “being moved.” Nevertheless, we use the vernacular term “being moved” in our measure as an operationalization of the feeling component of kama muta, because English speakers typically label their kama muta experiences “being moved or touched.”

#### Kama Muta and Collective Action by Disadvantaged and Advantaged Groups

Landmann and Rohmann formulated and tested their being-moved explanation of collective efficacy with groups that presumably consider themselves as the disadvantaged groups opposing powerful others (a mining company and the authorities). If correct, the model should generalize to other situations of social movements, including the BLM movement. However, does it also hold for advantaged groups?

We argue here that the model should apply to members of both advantaged and disadvantaged groups. As explained above, members of an advantaged group who perceive the situation to be unjust or as violating moral standards and are thereby motivated to change the situation, can identify with the disadvantaged group and/or adopt a politicized identity with a movement, which can cross the original intergroup divide. In both cases, perceiving that the group one identifies with is unified and has the potential to change the situation through joint action (i.e., collective efficacy) should lead to kama muta.

Regarding social movements, our assumption is that they are understood as relationships on a foundation of a perceived shared essence of having the same beliefs, values and goals. Such relations are conceptualized as communal sharing relations by relational models theory (Often they add hierarchical structure and coordinate some tasks according to equality matching) (Fiske, 1992). Some evidence suggests that appraisals of increased interpersonal closeness in communal sharing relations and communal sharing-based morality predict feelings of being moved (Seibt et al., 2017). For instance, the perception of a sudden intensification of communal sharing by observing or participating in a demonstration or hearing a meaningful speech related to shared values of fairness would evoke kama muta (Fiske, 2019).

Social movements and social activists seem to successfully evoke kama muta to motivate people to participate and act through messages of communal identity and justice (Pierre, 2019). In political movements, using kama muta increases the motivation to support and commit to a political cause when there is partisan communal sharing (Seibt et al., 2019). These findings suggest that kama muta may have the potential to foster support for a social cause and engagement for the goals linked to a politicized identity.

The process is nicely illustrated by a the following quote from the (White) host of a widely distributed podcast published in June 2020, days after the murder of George Floyd:

“My kids really wanted to protest. […] I live in an overwhelmingly White town. […] It was important to them, […] it was important to me. […] We went as a family down to the town hall […] and stood on a corner […] with signs saying: “Enough is enough,” “Black Lives Matter.” […] I had one of the four or five most moving […] experiences of my life. […] I went down there thinking that we would have trouble. […] I would say the positive response to our protest on this busy intersection ran about six, seven or eight to one from the most unexpected places. […] We had guys on […] Harleys […] putting their thumbs up, […] guys in […] huge pickup trucks […] cheering us on, […] two sheriff’s cars … give us this very subtle thumbs up. […] So my plea to you [listeners] is: … Get on the right side of history. […] Make a sign and protest.” Metcalf et al. (2020) on the Slate Culture Gabfest Podcast, June 10, 2020).

Here, protest for the cause of a disadvantaged group after a moral norm violation resulted in an experience of support by other members of the advantaged group labeled as moving, and included the belief that there is large support (efficacy), and further support (plea for others to join). As noted above, kama muta theory specifically predicts that intensifications of communal sharing relations lead to the emotion. The theory has yet to spell out in detail what such intensifications can be, but common examples include reunions, altruistic help, selfless acts of compassion, expressions of love and closeness, and manifestations or affirmations of social inclusion. Subjectively, such events appear to be experienced as increases in social closeness. Affirmation of moral values of the communal relation seems to be an additional route (Seibt et al., 2017). Experiences of collective efficacy may embody intensifications of communal sharing for various reasons: the realizations that one is a member of a large social category, that one’s membership is valued positively by others, and that one’s values are shared. Also, the experience of collaboration and coordination toward a shared goal based on the shared membership in a social group, or manifestations of communal bonds through various non-verbal channels such as synchrony and physical closeness (Fiske, 2004).

It is also possible that when perceiving injustice and moral norm violations, members of advantaged groups identify with the disadvantaged group. The same processes of experiencing collective efficacy should apply, possible through vicarious or third-person kama muta, and perhaps enhanced by compassion and empathic concern (Zickfeld et al., 2017).

Communal sharing relations largely neglect to keep track of contributions, but rather follow the principle of distributing resources according to need and ability. Once experienced, kama muta is theorized to result in increased motivation to affirm and engage in the same relation and to relate based on communal sharing principles. Extant studies on kama muta have found support for these ideas using self-reported general motivations (e.g., “I wanted to do something extra-nice for someone” in Zickfeld et al. (2019a), self-reported intentions to support a political candidate (“more inclined to vote for” and “motivated to work to help elect [the candidate],” Seibt et al., 2019). Other studies that measured self-reported being moved have also found supporting evidence. For instance, showing moving videos not only leads to self-reports of feeling moved and warm feelings in the chest, but also more actual helping behavior for an unrelated experimenter (Schnall et al., 2010) and more actual donations to charities (Sparks et al., 2019).

In sum, we propose that kama muta theory is consistent with the idea that collective efficacy experiences and beliefs give rise to experiences of being moved, and in turn strengthen devotion to and motivation to participate in collective action.

## The Current Research

Following these arguments, the present research investigates the link between injustice and collective efficacy appraisals on collective action. We specifically investigate the role of kama muta along with sadness and anger as positive correlates of collective action. Given that the United States has been the epicenter of massive mobilizations supporting racial equality in 2020/2021, we investigated participation in the BLM in this geographical context. We replicate and extend prior research on emotions and collective actions in several ways:

At the center of our study is a replication of Landmann and Rohmann (2020), where we test the hypothesis that two emotional pathways mediate between common appraisals and collective action: Anger is hypothesized to mediate between injustice appraisals and collective action, while being moved/kama muta is hypothesized to mediate between collective efficacy appraisals and collective action. We extend the contribution of this research by replicating the model in a non-environmental context that involves the interaction of advantaged and disadvantaged groups.

Secondly, given that understanding the emotional process involved in advantaged and disadvantaged groups is necessary to develop strategies to achieve social change, we collected data from both Black and White Americans; insights in this area are particularly relevant for cross-group helping and intergroup relations research.

Third, we exchange Landmann and Rohmann’s being moved measure (consisting of three feeling labels) for a measure designed based on kama muta theory that also includes feeling labels, and references to appraisals, physiological reactions, valence, and motivation (Coppin and Sander, 2021).

**FIGURE 1:**
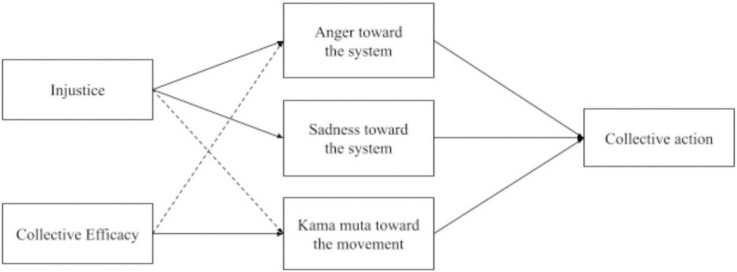
The mediation model with paths to collective action. Solid lines were hypothesized and confirmed. Dashed lines were found to be significant in exploratory tests.

Fourth, we collected data on multiple targets of those emotions, both concrete in- and outgroups and more politically defined social entities (the BLM, and the system of racial inequality). In line with the findings by Landmann and Rohmann (2020), we hypothesized that kama muta toward the BLM movement mediates the relationship between collective efficacy and collective action intentions. Additional hypotheses, including the role of anger and sadness, were also included (see Figure 1).

As a fifth goal, we use measures of identification with various groups and identities to understand the nature of the kama muta experiences that we observe. Landmann and Rohmann found the higher politicized identification (i.e., identifying with the environmental activist group) was, the more participants reported participating in effortful and non-normative collective actions. Here, we use the identification measures in exploratory analyses to understand how politicized identity relates to kama muta in disadvantaged and advantaged groups.

## Pilot Study

In the pilot study, we piloted a comprehensive measure of emotions and obtained the first exploratory data of the emotions and their association to collective action in the BLM movement. We targeted the measure of emotions toward the BLM movement, the system of racial inequalities, and Black and White people. We selected these targets because they represented the involved ethnic categories, the most active social movement, and the antagonist that this movement identified. We explored the associations between the emotions toward each target and their relationship with collective action.

### Method

#### Participants

We recruited *N* = 100 participants from Academic Prolific^1^ requesting US Americans. Participants were compensated with 1.25 GBP for approximately 10 min of their time. We excluded participants who failed 1 or both of the included attention checks and those who did not complete the survey (*n* = 22). The remaining 78 participants were 19–69 years old (*M* = 34.74, *SD* = 11.78); 32 reported being female, 45 male, and one third gender. Regarding ethnicity, 64.1% reported being White and the 35.9% remaining mentioned being members of ethnic minorities (Black *n* = 7, Asian *n* = 13, Hispanic *n* = 5, Other *n* = 3). Participants indicated their political ideology on a scale from 1 (“left”) to 10 (“right”); the average value was *M* = 4.60, *SD* = 2.73, 64.1% reported values between 1 and 5. Thus, the sample was skewed toward leaning politically left, and there were few Black participants.

#### Procedure

Each participant was presented with a survey delivered online through Qualtrics.com. First, participants read an information sheet and signed a consent form. Then, participants were asked to respond to the measures in the same order described below. Data analysis was performed using SPSS statistics version 26.

#### Materials

##### Emotions

The emotional experience in the past year was assessed through a new measure designed for this research. We presented participants with the descriptions of three emotions: sadness, anger, and kama muta. Descriptions contained detailed information about the valence, appraisals, physical reactions, and labels that characterize each one of those emotions. Here is an example of the description that was used to measure kama muta. Note that it starts off without naming any label. Because it is only our theoretical term, the term kama muta was never mentioned:

“This emotion gives you a positive feeling, and it makes you feel connected to others. Perhaps you have felt this emotion after experiencing a sudden sense of closeness to somebody else or an incredibly strong bond with another person or a group of people. When this emotion is mild, it feels a little warm and fuzzy. When it is more intense, you may notice warmth or a stirring in the center of the chest; your eyes may tear up, or you actually weep. Some people experience chills or goosebumps. You would probably call this as being touched or moved, and you would perhaps call the situation heart-warming.”

After each description, participants indicated their emotional experiences on four items measuring the frequency [“How often did you feel this emotion toward the (target) during the past year?”], intensity [“During the past year, how strongly did you typically experience this emotion toward the (target)?”], salience [“During the last year, how present was this emotion in your life, toward the (target)?”], and ease of retrieval [“How easily do situations from the last year come to your mind where you felt this emotion toward the (target)?”] of the respective emotion experienced.

The above items were used to measure each emotion (i.e., kama muta, sadness, and anger) toward each of the four targets: the system of racial inequalities, the BLM, and White and Black people. Items were answered on 5-point Likert scales ranging from 1 = *not at all/never* to 5 = *very much/always*. In total, we assessed each target with four items for each one of the three emotions in a 4 (targets) × 3 (emotions) design (see Supplementary Material for an overview of the full measure).

##### Collective Action

Participants reported their intentions to participate in collective action toward racial equality on a 5-point Likert scale ranging from 1 = *strongly disagree* to 5 = *strongly agree.* The seven items were adapted from Landmann and Rohmann (2020) and included: “I would/participate in a demonstration against racism, participate in a protest for racial equality, volunteer in an organization to fight racism, convince others to engage in racial equality discussions, sign petitions to stop racism, donate to a charity which supports Black people, join a campaign online to share information supporting racial equality.”

### Results

#### Internal Consistency of the Measures

We calculated Chronbach’s alpha for the four items for each emotion and target separately. Analyses demonstrated high internal consistency for all 12 scales. Subsequently, we averaged all items for each emotion and target and created composite variables. Reliability values and descriptive information for the scales are presented in Table 1. A correlation table with all variables can be found in the Supplementary Material.

**TABLE 1:** Reliability values and average levels of the emotions toward the four targets in the pilot study.

Scale	α	*M*	*SD*	*CI*
Anger toward System	0.91	3.08	1.09	[2.84; 3.33]
Anger toward BLM	0.95	2.31	1.24	[2.03; 2.59]
Anger toward Black	0.95	1.87	1.03	[1.63; 2.10]
Anger toward White	0.92	2.60	1.07	[2.36; 2.84]
Kama muta toward System	0.96	1.75	1.03	[1.51; 1.98]
Kama muta toward BLM	0.95	2.48	1.21	[2.21; 2.75]
Kama muta toward Black	0.93	2.91	1.01	[2.68; 3.14]
Kama muta toward White	0.93	2.60	1.06	[2.37; 2.83]
Sadness toward System	0.96	3.27	1.25	[2.99; 3.55]
Sadness toward BLM	0.93	2.31	1.09	[2.07; 2.56]
Sadness toward Black	0.93	2.32	1.07	[2.08; 2.56]
Sadness toward White	0.94	2.57	1.12	[2.31; 2.82]
Collective action	0.94	3.54	1.18	–

*Means and 95% confidence intervals; System refers to system of racial inequality, BLM refers to Black Lives Matter Movement, Black refers to Black people, White refers to White people.*

#### Emotions Toward Different Targets

To investigate the differences between emotional reactions to different targets, we conducted an ANOVA with the target (system vs. movement vs. Black people vs. White people) and emotion (anger vs. kama muta vs. sadness) as within-participant factors. Mauchly’s test indicated a violation of sphericity assumption χ^2^ (20) = 117.92, *p* < 0.001. Since sphericity was violated (ε = 0.660), Greenhouse–Geisser-corrected results are reported. Results indicate that there was no overall difference between emotions *F*(1.88,144.66) = 2.367, *p* = 0.101, η^2^ = 0.03. However, there were differences between targets irrespective of the emotions *F*(2.53,194.80) = 6.500, *p* = 0.001, η^2^ = 0.07. Finally, the emotions and target factors interacted significantly *F*(3.96,304.92) = 29.79, *p* < 0.001, η^2^ = 0.28. The system of racial inequalities elicited the highest levels of anger and sadness, while Black and White people elicited the highest levels of kama muta (see Table 1).

#### Exploratory Analysis on the Relationship of Collective Action and Emotions

In order to explore the association between the emotions, kama muta, sadness, anger, and collective action, we calculated a multiple linear regression. The model was run as a stepwise regression with the default settings in SPSS, which includes predictors with *p* < 0.05 and excludes them with *p* > 0.10 iteratively. We included the 12 composite variables as potential predictors. Additionally, we examined whether ethnicity could have an impact on the model and split the sample into White and non-White participants based on their reported ethnic identity. Table 2 summarizes the variables that significantly predicted collective action. Kama muta toward the BLM movement was a consistent and strong predictor, as was sadness about racial inequality.

**TABLE 2:** Summary stepwise regressions pilot study.

	Total sample			White participants (*n* = 50)			Non-white participants (*n* = 28)		
	*R* ^2^	*p*	*F*	*R* ^2^	*P*	*F*	*R* ^2^	*p*	*F*
	**62.3**	**<0.001**	**40.70**	**67.2**	**<0.001**	**31.48**	**54.1**	**<0.001**	**14.72**

**Predictor**	**B**	**CI**	***p***	**B**	**CI**	***p***	**B**	**CI**	***p***

Kama muta to BLM	0.67	[0.51,83]	<0.001	0.66	[0.46, 87]	<0.001	0.57	[0.33, 80]	<0.001
Sadness to System	0.34	[0.20,48]	<0.001	0.32	[0.13, 51]	<0.001	0.29	[0.05, 53]	0.020
Kama muta to Black	–0.20	[–0.40,00]	<0.001						
Kama muta to White				–0.26	[–0.46,–0.05]	0.016			

*95% confidence intervals. Blank cells indicate that the stepwise regression removed this predictor from the equation. System refers to system of racial inequality, BLM refers to Black Lives Matter Movement, Black refers to Black people, White refers to White people.*

### Discussion

This study aimed to pilot a new measure of emotions and carry out exploratory analysis of the association of those emotions and collective action intentions. These findings indicate that the measure designed for the current research context satisfactorily differentiated the emotions for each target. The results of exploratory regression analysis revealed that kama muta toward the movement was positively associated with collective actions toward racial equity, followed by sadness toward the system of racial inequalities in the total sample and for each sub-group. Neither anger toward the movement nor anger toward the system of racial inequalities was significantly associated with collective action. Notably, while kama muta toward Black people was high overall, it correlated much less with collective action than kama muta toward the BLM movement did. In the stepwise regression, it even ended up with a negative sign (likely the outcome of a suppression effect).

These initial findings mainly supported our initial theorizing on the positive relationship between kama muta and collective action and sadness and collective action. Different from our theorizing, the relationship between anger and collective action was not statistically significant in the present sample. However, the pattern of results points to the expected direction. Notably, the sample size was small. Given the partial preliminary support for the hypothesized association of emotions and collective action, we set up the main study as a confirmatory test of our full theoretical model (see Figure 1).

## Main Study

The main study tested a mediation model to explain the relations between appraisals and collective action intention toward racial equity through the three emotions. To test this model, we surveyed only White and Black Americans. Specifically, we hypothesized that (H1) Kama muta toward the BLM movement mediates the relationship between collective efficacy and collective action; (H2) Anger toward the system of racial inequalities mediates the relationship between injustice appraisals and collective action intentions and (H3) Sadness toward the system of racial inequalities mediates the relationship between unfairness appraisal and collective action (see Figure 1). In addition, we explored whether the hypothesized model holds true for both the disadvantaged and advantaged group members that are Black and White people in the present context, and we also explored correlations with identification with the activist group (as in Landmann and Rohmann (2020)), the most the most relevant social movement (Black Lives Matter), the superordinate group (US Americans), and the advantaged and the disadvantaged group (White people and Black people). Preregistration of this study can be found here: *https://aspredicted.org/pd2w3.pdf*.

### Methods

#### Participants

We recruited *N* = 306 participants from Academic Prolific with an equal sampling of US Black and White Americans. We aimed to collect data for 300 participants, 150 participants in each group, given that correlations are more stable around this sample size (Schönbrodt and Perugini, 2013). Participants were compensated with 1.25 GBP for approximately 10 min of their time completing the survey. Based on our pre-registered exclusion criteria, we excluded participants if their response time exceeded 3 *SD* (*n* = 7) and if they failed any of the included attention checks (*n* = 83). The analyzed sample included *N* = 215 participants.

White participants (*n* = 107) were 18–70 years old (*M* = 34.57, *SD* = 12.69); 41 reported being male, 62 female and 4 third gender. Participants indicated their political ideology on a scale from 1 (“left”) to 10 (“right”); the average value was *M* = 3.40 (*SD* = 2.60). 84.1% reported at least some college education. Black participants (*N* = 108) reported age varying from 18 to 68 (*M* = 35.40, *SD* = 11.71), 57 reported being male and 51 female. The average value of ideology was 4.31 (*SD* = 2.49), and 84.3% reported some college education.

#### Procedure

Each participant had to answer a survey delivered online through Qualtrics.com. All participants completed the same questionnaire and answered the measures in the order described below. Participants indicated to what extent they agreed with a set of statements on collective efficacy and injustice, their emotional ratings toward two targets: the system of racial inequalities and the BLM movement, and their intentions to participate in collective action. The questionnaire ended with some exploratory variables (group attitudes, contact, and social identification, see Supplementary Material for a full list of included items).

#### Measures

##### Collective Efficacy and Injustice Appraisals

Appraisals were adapted from Landmann and Rohmann (2020). Collective efficacy appraisals were assessed with three items on a 5-point Likert scale from 1 = *not at all t*o 5 = *completely;* “I feel that together people can reduce racial inequality; I believe that together people can stop unfairness toward Black people; People can together, through joint effort, achieve racial equality.” Injustice appraisals were also measured with three items: “Racial inequality is unethical; Racial inequality violates moral rules; Discrimination toward Black people is unfair.”

##### Emotions: Kama Muta, Sadness, and Anger, and Collective Action

We assessed emotions as in the pilot study. The only difference was that we only collected data of the three emotions targeting the system of racial inequalities and the BLM Movement. Collective action was assessed as in the preliminary study, except that the items were on a 7-point Likert scale rather than a 5-point Likert scale.

##### Identification

Identifications were measured through the single-item social identification measure (SISI, Postmes et al., 2013). Participants had to indicate the level of agreement with “I identify with (BLM/US Americans/Racial Justice activists/White people/Black people)” on 7-point Likert scales from 1 *fully disagree* to 7 *fully agree*.

### Results

#### Internal Consistencies

The items for the appraisals, emotions, and collective action were analyzed for reliability. The scales were found to be highly reliable. Subsequently, we created composite variables by averaging the items for each appraisal, for each emotion and target, and for collective action. Descriptive statistics and reliability for each measure can be seen in Table 3. Correlations between all variables for the total sample and each group separately can be found in the Supplementary Material.

**TABLE 3:** Reliability values and descriptive for each scale and each group in the main study.

Scale	α	*M*	*SD*
Collective efficacy	0.95	4.05	0.90
Injustice	0.85	4.63	0.69
Anger to System	0.91	3.52	1.05
Anger to BLM	0.96	2.46	1.36
Kama muta to System	0.96	1.83	1.19
Kama muta to BLM	0.94	2.93	1.16
Sadness to System	0.94	3.52	1.11
Sadness to BLM	0.96	2.48	1.23
Collective action	0.93	5.24	1.52

*All items are 5-point Likert scales from 1 to 5, except collective action, which is in a 7-point Likert scale from 1 to 7. System refers to system of racial inequality, BLM refers to Black Lives Matter Movement, Black refers to Black people, White refers to White people.*

#### Emotions Toward the Targets

In order to provide some context for the emotion levels, we compared the levels of the three emotional reactions to the system of racial inequalities and the BLM movement. We conducted a GLM analysis with emotions and targets as within-participants factors and ethnicity as a between-group factor. For the interaction between emotion and target, sphericity was not met as indicated by Mauchly’s test, χ^2^(2) = 134.64, *p* < 0.001. Since sphericity was violated (ε = 0.69), Huynh-Feldt results are reported. We found main effects of emotion, ethnicity, and targets. More importantly, emotions differed depending on the target, as indexed by an emotion by target interaction, *F*(1.37,292.36) = 153.33, *p* < 0.001, η_*p*_^2^ = 0.42. However, there was no three-way interaction between emotion, target and ethnicity, *F*(1,37,292.36) = 1.726, *p* = 0.189, η_*p*_^2^ = 0.01. Finally, the test of between-subjects effects indicated that across emotions both groups differed in their overall emotion levels, *F*(1,213) = 12.73 *p* < 0.001, η_*p*_^2^ = 0.06. Black participants reported higher levels of emotional reactions across targets. The system of racial inequalities elicited a higher level of anger and sadness. The BLM movement elicited higher levels of kama muta, especially in Black participants (see Table 4).

**TABLE 4:** Mean values for the emotional reactions for each target and each group in the main study.

	White participants		Black participants	
	System	Movement	System	Movement
Anger	3.39 [3.18; 3.60]	2.13 [1.89; 2.38]	3.65 [3.46; 3.84]	2.80 [2.54; 3.07]
Kama muta	1.56 [1.37; 1.75]	2.93 [2.77; 3.08]	2.09 [1.84; 2.34]	3.13 [2.90; 3.37]
Sadness	3.48 [3.28; 3.68]	2.37 [2.15; 2.59]	3.56 [3.34; 3.78]	2.59 [2.35; 2.84]

*Means and 95% confidence intervals.*

#### Hypothesis Testing

We conducted first regression analyses and then structural equation modeling to test our hypotheses. The regressions are to some extent redundant with the structural models. However, we report here both because the regressions isolate the differences between the groups better.

##### Collective Action Regressed on Appraisals

We hypothesized that (H1.a) Collective efficacy appraisals are positively associated with collective action intentions and (H2.a) Injustice appraisals are positively associated with collective action. We ran a multiple regression on the complete sample while including ethnicity as a moderator. As pre-registered, we regressed collective action on ethnicity (contrast-coded as –1/2 for White participants and +1/2 for Black participants), injustice appraisal (centered), collective efficacy (centered), and the interaction terms of both injustice and efficacy with ethnicity. Note that we did not include the three-way interaction. Also, note that centering was done on the complete sample.

The model explained 27.4% of variance of collective action, *R*^2^ = 0.27, *F*(5,209) = 15.80, *p* < 0.001. The intercept was *B* = 5.25, and represents the average of collective action scores. The main effect of ethnicity was not significant, *B* = –0.03, *p* = 0.885. Both appraisals explained a significant amount of variance. Injustice appraisals were positively associated with collective action, *B* = 0.57 [0.30,0.84], β = 0.26, *p* < 0.001. Collective efficacy appraisals were also positively associated with collective action, *B* = 0.63 [0.42,0.84], β = 0.37, *p* < 0.001. Ethnicity did not moderate the effect of injustice, *B* = –0.34 [–0.89,0.20], β = –0.08, *p* = 0.212 and neither the effect of collective efficacy, *B* = –0.29 [–0.71,0.13], β = –0.09, *p* = 0.171. Analysis run separately in each group can be found in the Supplementary Material.

##### Collective Action Regressed on Emotions

**FIGURE 2:**
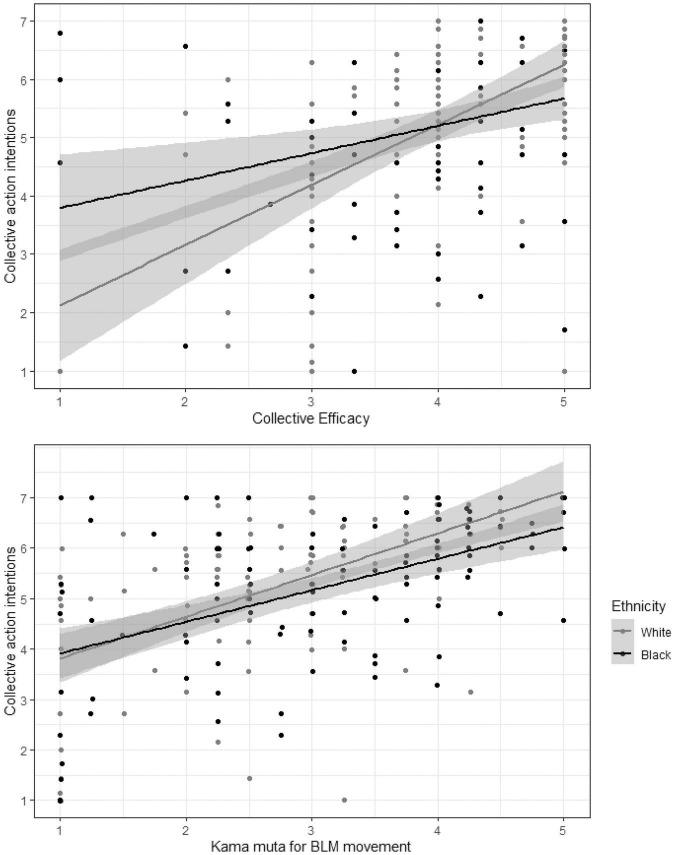
Scatterplots of the relationship between collective efficacy and collective action and Kama muta for BLM and Collective action intentions for both Black and White people. Gray areas show 95% Confidence Intervals.

The model explained 54.5% of the variance of collective action *F*(7,207) = 35.36, *p* < 0.001. The intercept was *B* = 5.23. The main effect of ethnicity was marginally significant, indicating slightly higher values for white *B* = –0.32 [–0.61, –0.03] compared to black participants, *B* = –0.11, *p* = 0.030. In line with hypotheses, the three emotions were positively associated with collective action intentions: Anger, *B* = 0.67 [0.49,0.85], β = 0.46, *p* < 0.001, kama muta, *B* = 0.32 [0.18,0.47], β = 0.25, *p* < 0.001, and sadness *B* = 0.10 [0.02,0.38], β = 0.15, *p* = 0.023. Ethnicity did not moderate the effect of anger, *B* = 0.12 [–0.24,0.48], β = 0.52, *p* = 0.523, and neither kama muta, *B* = 0.17, β = 0.07, *p* = 0.245 [–0.12,0.47] (see Figure 2). However, ethnicity moderated the effect of sadness, *B* = –0.55 [–0.90, –0.20], β = –0.20, *p* = 0.002.

We proceeded to analyze the model in each group separately without centering variables. The model explained 61.4% of the variance of collective action for White participants *F*(3,103) = 54.50, *p* < 0.001 and 46.2% of the variance for Black participants, *F*(3,104) = 29.82, *p* < 0.001. For White participants, anger, *B* = 0.61 [0.35,0.88], β = 0.41, *p* < 0.001, sadness *B* = 0.48 [0.19,0.76], β = 0.32, *p* = 0.001, and kama muta, *B* = 0.24 [0.01, 46], β = 0.16, *p* < 0.001, were positively associated to collective action. For Black participants, anger *B* = 0.73 [0.49,0.97], β = 0.50, *p* < 0.001 and kama muta *B* = 0.41 [0.22,0.60], β = 0.34, *p* < 0.001, but not sadness, *B* = –0.07 [0.19,0.76], β = –0.74, *p* = 0.460, were positively associated to collective action intentions. For both groups anger explained most of the variance of collective action. Kama muta descriptively explained more variance in the Black participants than in White participants, but this was not statistically significant, as noted above. Sadness significantly explained variance in White participants but not in Black participants.

##### Structural Equation Modeling

**FIGURE 3:**
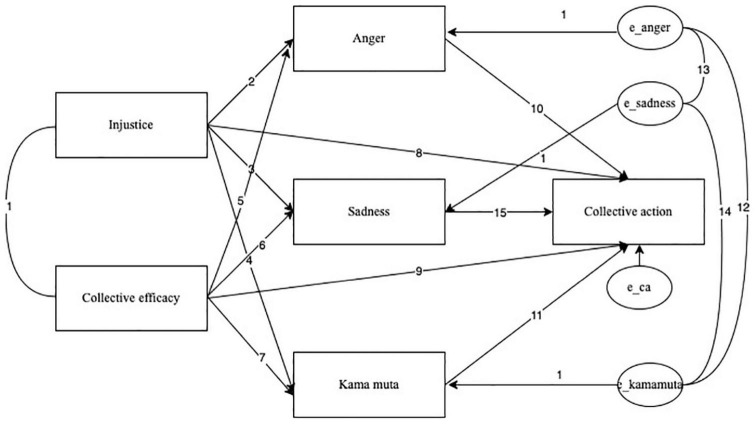
Path model with all the interactions and parameters.

We performed a Structural equation modeling (SEM) to test the mediation model, using SPSS Amos version 27. The model combined the analyses above. It featured two independent variables (injustice and collective efficacy appraisals), the three emotions as mediators, and collective action as the dependent variable. The complete model is depicted in Figure 3. The independent variables were allowed to correlate, as well the residuals of the three emotions. Direct effects of the appraisals on collective action were also included. The overall fit was examined using the chi-square test and the root mean square error of approximation (RMSEA). A bootstrapping method (5000 iterations) was used to test the statistical significance of mediation with all mediators in the model. A multigroup analysis was performed to examine whether the mediation model was moderated by ethnicity. To do that, all regression weights and covariances were labeled in the model. There were in total 15 of them.

A preliminary model (1) tested whether the covariances among the residuals of the emotion terms (12, 13, 14) could be set to zero while allowing all other parameters to differ between the groups. This model did not fit the data well, RMSEA = 0.286 [0.24,0.33], χ^2^(6) = 110.29, *p* < 0.001. These parameters were thus allowed to differ from 0 in all following models. This indicates that, unsurprisingly, the correlations among the three emotions cannot be fully explained by the two appraisals.

In a second model, we set all parameters to be equal in the two groups (i.e., b1 = w1, b2 = w2, b15 = w15). This tested the hypothesis that the two groups do not differ regarding the relationship between the variables. Overall fit was not good, RMSEA = 0.104 [0.07,0.14], χ^2^(15) = 49,51 *p* = 0.003. We inspected all modification indices > 2 (see Supplementary Material). The modification indices indicated that relaxing the following parameters would substantially improve the fit of the model: (1) Covariance between unfairness and collective efficacy, (2) Regression path between collective efficacy and sadness, and (3) Regression path between sadness and collective action (which is in line with the regression analyses). In addition, the modification indices suggested adding a regression between kama muta and sadness in this model, which we did not follow, given that the residuals of the two emotions are allowed to correlate. In sum, the second model showed that Black and White participants differed regarding the covariance of the two appraisals and the influence of collective efficacy on collective action *via* sadness.

Consequently, in model 3, we allowed parameters 1, 6, and 15 to differ between the groups, while all other parameters were set to be equal. The overall fit is good, RMSEA = 0.044 [0.00,0.09], χ^2^(12) = 16.86, *p* = 0.540 (MacCallum et al., 1996). All parameters reported are from this model. Table 5 shows the summary of the coefficients for both groups separately.

**TABLE 5:** Summary of multigroup path analysis.

Path	White participants				Black participants			
	*B*	*SE*	CR	β	*B*	*SE*	CR	β
Injustice→anger	0.57	0.09	6.24***	0.41	0.57	0.09	6.24***	0.34
Collective efficacy →kama muta	0.30	0.08	3.58***	0.24	0.30	0.08	3.58***	0.23
Injustice→sadness	0.26	0.10	2.60 **	0.19	0.26	0.10	2.60†	0.14
Injustice→kama muta	0.36	0.11	3.40***	0.25	0.36	0.11	3.40***	0.19
Collective efficacy → sadness	0.51	0.09	5.51***	0.43	–0.08	0.11	–0.65†	–0.06
Collective efficacy → anger	0.30	0.07	4.16***	0.24	0.30	0.07	4.15***	0.26
Injustice→ collective action	0.14	0.11	1.23†	0.41	0.14	0.11	1.23†	0.06
Collective efficacy → collective action	0.30	0.09	3.48***	0.16	0.30	0.09	3.48***	0.19
Kama muta → collective action	0.31	0.07	4.36***	0.21	0.31	0.07	4.36***	0.26
Anger → collective action	0.61	0.09	6.79***	0.40	0.61	0.09	6.79***	0.44
Sadness → Collective action	0.28	0.11	2.46†	0.18	0.01	0.09	0.06†	0.01

***p < 0.001, **p < 0.05, †p < 0.1.

*Anger refers for anger toward the system of racial inequality, kama muta refers to kama muta toward the Black Lives Matter movement, and sadness refers to sadness over the system of racial inequality. CR refers to critical ratio.*

Injustice and collective efficacy appraisals covaried for White participants (*r* = 0.39, *p* < 0.001), but not for Black participants (*r* = –0.06, *p* = 0.588). The standardized total effect of collective efficacy on collective action, which includes both mediated and direct effects, was significant, for both White, β = 0.38, *p* < 0.001, and Black, β = 0.37, *p* < 0.001, participants. The standardized total effect of injustice on collective action, which includes both mediated and direct effects, was significant for both White, β = 0.31, *p* < 0.001, and Black participants, β = 0.26, *p* < 0.001. The standardized indirect effect of collective efficacy through emotions on collective action is β = 0.22 for White and β = 0.18 for Black participants. The mediated effect of injustice through emotions on collective action is β = 0.25 for White and β = 0.20 for Black participants. This indicates that more than half of the total effect of collective efficacy and two-thirds of the effect of injustice on collective action is mediated through emotions.

After adjusting the model, the SEM findings reveal that the direct path from injustice to collective action was not significant anymore. Collective efficacy, however, retained a significant direct path to collective action. It was also found that collective efficacy was significantly related to kama muta, sadness, and anger. Kama muta and anger, but not sadness were significantly associated with collective action (see summary of multigroup path analysis in Table 5).

A bootstrapping method was used to judge the significance of specific indirect effects of injustice and collective efficacy through each emotion on collective action. The mediation hypotheses were tested by requesting an additional estimate from Amos. The predicted meditations were tested with an alpha level of 0.05 each. In addition, we defined a new alpha level for the six unpredicted mediation tests as these were exploratory (in the lower half of Table 6). Bonferroni correction equals a new alpha level of 0.05/6 = 0.008. All mediation estimates are depicted in Table 6.

**TABLE 6:** Main indirect effects in the structural equation model.

Mediation	*B*	*p*	*CI*
Injustice via anger	0.344	< 0.001	[0.22; 52]
Collective efficacy via Kama muta	0.091	0.001	[0.04;0.17]
Injustice via Sadness (Black)	0.001	0.896	[–0.04;0.05]
Injustice via Sadness (White)	0.071	0.030	[0.01;0.18]
Collective efficacy via anger	0.179	<0.001	[0.09,0.30]
Injustice via kama muta	0.110	0.001	[0.05,0.19]
Collective efficacy via Sadness (Black)	0.000	0.779	[–0.02,0.02]
Collective efficacy via Sadness (White)	0.142	0.033	[0.03,0.28]

*All paths lead to Collective action. 95% confidence intervals.*

The results revealed that as hypothesized, kama muta mediated the relationship between collective efficacy and collective action, and anger mediated the relationship between injustice and collective action. In addition, sadness mediated the relationship between injustice and collective action, but only for White participants.

Finally, in the exploratory part, anger also mediated the relationship between collective efficacy and collective action, and kama muta mediated the relationship between injustice and collective action even after Bonferroni correction. In sum, both anger and kama muta mediated the relationship between both appraisals and collective action, while sadness only mediated the association between injustice and collective action for white participants.

#### Exploratory Analyses: Correlations With Identification

We assessed the association between the main variables of the study (kama muta toward the movement and collective action) and the social identification options for each group separately (see Supplementary Material). For Black participants, collective action motivation was most strongly associated with identification with Racial Justice activists (*r* = 0.77), the BLM (*r* = 0.59), and Black people in general (*r* = 0.48). The other possible identifications play no role. Kama muta toward the movement was associated most with identification with the movement (*r* = 0.60), somewhat less with identification with racial justice activists (*r* = 0.53), and still less with identification with Black people (*r* = 0.27). Collective efficacy is linked to identification with racial justice activists (*r* = 0.37) and BLM (*r* = 0.32), and surprisingly also somewhat with identification with White people (*r* = 0.23). (All numerically listed correlations were statistically significant, but note that we did not correct for multiple comparisons).

For White participants, collective action motivation was most strongly associated with identification with both Racial Justice activists (*r* = 0.73) and the BLM (*r* = 0.72), and less but still significantly with Black people in general (*r* = 0.32). The other possible identifications played no role. Kama muta toward the movement in White participants was associated with identification with both racial justice activists specifically (*r* = 0.43) and the BLM in general (*r* = 0.41), and less but still significantly with identification with Black people (*r* = 0.21). Collective efficacy is again linked to identification with racial justice activists (*r* = 0.45) and BLM (*r* = 0.41), and this time also with identification with Black people (*r* = 0.32). (Again, all numerically listed correlations were statistically significant).

### Discussion

The main study tested how the effects of appraisals of injustice and appraisals of collective efficacy on collective action were mediated by emotions of anger, kama muta, and sadness. The regression analysis confirmed that both collective efficacy and injustice appraisals were positively associated with collective action intentions in the full sample. Additionally, the three emotions were predictors of collective action intentions toward racial equity in the full sample, yet, after additional analyses by group, sadness was a predictor only for White participants. The structural equation modeling allowed a multigroup comparison of the mediation models between White and Black participants. As predicted, the effects of injustice and collective efficacy appraisals on collective action were mediated by anger and kama muta, respectively, in both White and Black participants. In addition, we found the respective other mediations in exploratory analyses. Exploration also showed that Black and White participants differ in the covariance of the two appraisals and in sadness *via* collective efficacy path. Collective efficacy and injustice were associated with sadness, but these associations and the mediation role of sadness for injustice were significant only for White participants.

The exploratory correlation analyses of the identification measures showed that for Black participants, collective action, identification with both the BLM movement and racial justice activists, and kama muta toward the BLM movement are all closely associated. Collective efficacy is also tied to identification with both BLM and activists, but less strongly. For White participants, the picture looks similar but more differentiated. On the one hand, collective action is correlated even more strongly with identification with the BLM movement and also identification with racial justice activists. Kama muta with BLM is clearly correlated with identification with BLM and activists, but somewhat less than for Black participants. Collective efficacy is also tied to identification with both BLM and activists for White participants.

## General Discussion

The current research investigated the role of kama muta for collective action intentions toward racial equity in the context of the BLM. We hypothesized that kama muta toward the BLM movement would mediate the path of collective efficacy appraisals to collective action intentions. In addition, we included hypotheses about the role of anger and sadness toward the system of racial inequalities as mediators between injustice appraisals and collective action intentions.

### Findings

In the pilot study, we found that kama muta toward the movement and sadness toward the system were the main predictors of collective action intentions toward racial equity. In the main study, we found that kama muta toward the movement mediated the relationship between collective efficacy and collective action for both Black and White participants. Anger toward the system of racial inequalities fully mediated the relationship between injustice and collective action. Unexpectedly, we found that anger also partially mediated the association between collective efficacy and collective action, and kama muta partially mediated the association between injustice and collective action. In addition, we discovered that sadness toward the system of racial inequalities mediated the relationship between unfairness and collective action intentions; however, this effect was only significant for White participants. Finally, identification with both BLM movement and racial justice activists was correlated (a) strongly with collective action, (b) solidly with kama muta with the BLM movement, and (c) also solidly with collective efficacy in both Black and White participants. Kama muta with the movement was correlated more clearly with identification with the movement in Black participants than in White participants.

Thus, the results confirmed the mediating role of kama muta on the relationship between collective efficacy and collective action. Nonetheless, it also indicates a mediator role of kama muta for the effect of injustice. In addition, the model held for both White and Black participants, meaning that there was no difference between both groups in the pathways to collective action. It is notable that injustice appraisals and collective efficacy were correlated for White but not Black participants.

The findings on kama muta conceptually replicate and extend the findings that Landmann and Rohmann (2020) reported for activists who opposed a mining company and their sympathizers. The current findings show that these predictions hold when (1) a different measure is used (based on kama muta theory and including references to appraisals, feeling labels, bodily sensations, and motivation, rather than based on an approach focusing on being moved in general, and using feeling labels only), (2) when conducted in English rather than German, and (3) when tested in a different context than environmental actions, (4) and perhaps most importantly, for both the disadvantaged and the advantaged group in a struggle that aims at reduction of social inequalities. The results also demonstrate that kama muta is clearly different from sadness, which showed a separate path that was only present for the advantaged group.

### Limitations and Future Directions

#### Measures and Sample

Any research on emotions, especially when it uses self-report on longer periods of time, is limited by participants’ ability to remember, and verbally report the emotions. Discussing these measures critically is thus crucial. The current work based the design of the emotions measure and the general theoretical argument on the kama muta construct rather than on Landmann and Rohmann’s *being moved* construct, but we reiterate here that this distinction is largely theoretical for the present results. The two theories differ mainly in an area that was included in neither study, namely their conjectures about *why* situations that cause the constellation of kama muta or being moved do that. While there is an overlap in the causes assumed by both theories (e.g., exceeded expectations of prosociality), there are also diverging ideas (see section “Introduction” and also below). This would clearly be a fruitful area for further work.

The kama muta measure designed and used in the current studies was piloted but otherwise not validated. It includes a more complete description of emotional experiences that can be directed to different targets. We are confident that is an appropriate measure because (a) it was based on a well-researched theoretical background, and (b) because the equivalent (and also new) measure of anger replicated the classic mediation between injustice and collective action. Nevertheless, future research should validate this measure of kama muta and adapt it to other intergroup contexts. Note that the design of the measure presented a conundrum. We opted for giving a rich description of the kama muta prototype that included an abstract description of the appraisal and the resulting motivation. The prompt was written in a way that asked participants to compare emotional experiences in the last year to this abstract description. Nevertheless, there is a potential downside to this richness, namely that the kama muta measure captures variance that actually belongs to predictor (collective efficacy) and outcome (motivation). We acknowledge this as a shortcoming of the study, but are confident that it does not compromise the results given that many other studies with separate measures of the kama muta components typically find parallel predictions by all components (with physical symptoms and motivation typically performing least well). The fact that our results closely parallel those of Landmann and Rohmann (2020), who relied on feeling labels alone, support this.

It is important to mention that the sample in this study is not representative. Larger and more representative samples could provide further evidence of the stability of these results. In addition, the results surely capture a transient moment in the life of a social movement. The items asked participants to reflect on their last year, which included massive protests, a polarizing presidential election with campaigns that often referenced the movement, and a pandemic that highlighted tragic vulnerabilities caused by the same social inequalities.

#### Test of Theoretical Model

In general, more evidence is needed to test the causality of the association between emotions and appraisals, therefore experimental research could provide insights in this direction. Note, however, that the kama muta model to some extent predicts reciprocal effects, especially over time in a dynamic social relation. If people experience kama muta from seeing joint demonstrations of advantaged and disadvantaged groups for values that they support and then join a demonstration themselves, this will give them another boost of experiencing collective efficacy and encourage further kama muta experiences.

The current data do not yet identify what elements of experiencing collective action lead to kama muta experiences and what role morality plays. Qualitative data could add more depth to understanding the context in which this emotion arises in a movement like Black Lives Matter. The idea that experiencing collective efficacy is equivalent to a communal sharing intensification and thus leads to kama muta experiences may be surprising at first. However, imagine situations like joining together to help a child that got lost, pushing a car out of mud, or defeating an opposing team in a tug-of-war. The experience of jointly having the potential and indeed power to enact collective action might act as a manifestation and confirmation of the communal sharing relation and thereby cause an experience of oneness and togetherness (Fiske et al., 2019), which can, in turn, become the source of kama muta.

In addition to the mediator role of kama muta through the path of collective efficacy, the results showed that kama muta also mediated the association between injustice and collective action. This relation was not predicted, but strong enough to survive the correction for multiple comparisons for the unpredicted mediations, and it deserves to be taken seriously while being interpreted with caution. Similar to our results, Landmann and Rohmann (2020) found that the feeling of being moved mediated the association between injustice appraisals and collective action. These authors suggest that people were “negatively moved” by unfair practices. Our results indicate that sadness does not account for the collective action of the disadvantaged group. It could be possible that appraisals of injustice could elicit kama muta toward the movement because the movement represents positive core values such as fairness and equality (Cova and Deonna, 2014) that are experienced as shared and may be necessary to contrarest the unfairness. Likewise, the observation that anger mediates the relationship between collective efficacy and collective action makes sense when considering that anger arises when one appraises a potential to change the situation (Roseman, 2013). In light of such speculations, it becomes clear that better measures of experienced or observed intensification of communal sharing are needed to test kama muta theory comprehensively.

Nevertheless, these results contrast with most collective action models that emphasize the tight association between injustice appraisal and anger, indicating only *one* emotional path to collective action. There might in fact be other emotions that play a role. Landmann and Rohmann (2020) pointed out collective guilt as a potential contributor (Ferguson and Branscombe, 2014), and we agree that collective guilt can shape not just engagement in, but also the specific goals of collective action (Masson and Fritsche, 2021). However, guilt is experienced mainly by the advantaged group (Thomas et al., 2009b). Therefore, guilt might indeed be involved in the effect of sadness in White participants in the current study. Finally, another interesting avenue for future work might be emotions coming out of the intensification of other relational models, such as authority ranking – which might indeed be a part of what is commonly labeled as awe.

#### Kama Muta and Politicized Identities

Being moved and kama muta are conceptualized as social-relational emotions that are influenced by, and themselves result in, relational processes. The current work focused on the larger picture, from judgments of the social situation (injustice, collective efficacy) to collective action. Disentangling the more minute details, including tracing how social identification with the various identities involved change throughout these emotional experiences, remains a task for future work. However, the current research offers some insights in how kama muta (and also anger) and different social identities interact in the context of collective action.

We started our exploration in the pilot study by focusing on both politicized identities (BLM movement, “system of racial inequality”) and ethnically defined identities (Black and White people). The former outperformed in the prediction of collective action, and thus, we focused on politicized entities. This is in line with longstanding arguments and findings that politicized identities play a major role in social change (van Zomeren et al., 2018). In the main study, the correlations between kama muta and the various social identification measures show that kama muta for the movement is indeed associated with politicized identities (BLM and racial justice activists) for both White and Black participants. One way to interpret this is to say that by fostering politicized identities, kama muta and other relationship processes could allow the creation of communal sharing relationships that would enable advantaged and disadvantaged group members to work together toward a common cause. But two caveats are necessary. First, reciprocal relations between identification, collective efficacy, and kama muta are likely. Such reciprocal relations with identification have been pointed out before (Kessler and Hollbach, 2005; van Zomeren et al., 2008), and experimental and longitudinal work is necessary to chart them.

Second, the correlations that we observe tentatively suggest a more central, or perhaps more uniform, role for kama muta toward the movement for Black participants than for White participants. It is possible that there is more diversity in what moved White participants about the BLM movement, and what identifications are at play. Some may see BLM as a movement that bridges the disadvantaged and advantaged group and be moved by the collective efficacy of this new joint social group that they identify with. A politicized collective identity based on shared values and beliefs about fairness and equity seems promising to integrate advantaged and disadvantaged groups (Subašić et al., 2008; Thomas et al., 2009a). Others may be moved by experiences of collective efficacy in their own in-group when protesting moral violations, but not identify with a movement that bridges the groups (and instead identify with a subgroup of their advantaged in-group). This is in line with arguments by Craig et al. (2020), who suggest that advantaged groups take actions based on values and norms rather than shared identification, and that perception of privileges is essential to encourage action. These possibilities reveal a weakness of the collective efficacy measures, which simply referred to “people,” rather than specifying any exact group. The measures of collective efficacy, kama muta, and identification did thus not perfectly align. This has the advantage of making comparison to earlier studies easier, but the disadvantage of obscuring the actual identities involved. Future work could use more targeted measures. Due to the likely reciprocal processes and this ambiguity, we only presented correlational analyses rather than placing identification in the causal model.

It is noteworthy that we found no association between US American identity to kama muta toward the movement or to intentions to participate in collective action. Previous work has shown that kama muta can reduce affective polarization and increase the salience of a common in-group in the context of US politics (Blomster Lyshol et al., 2022). Common in-group identity can promote the reduction of stereotypes, prejudice, and discrimination toward the out-group (Dovidio et al., 2000, 2010). However, it can undermine collective action intentions (Ufkes et al., 2016). Banfield and Dovidio (2013) found that emphasizing a common “American” identity in White participants reduces the recognition of discrimination against Black people and consequently also their willingness to support them. In consequence, the authors suggested that awareness of racial injustice is essential to encourage collective action (see also Uluğ and Tropp (2021)).

Based on the context of the BLM, we have mainly focused on collective action that is aimed at increasing equality and intergroup relations. Note, however, that the exact same emotional processes should propel collective movements aimed at other goals. Fiske (2019) argued that it is likely that participants of fascist rallies in Nazi Germany fostered a sense of commonality and kama muta among its participants. Seibt et al. (2019) found that both Republicans and Democrats experienced kama muta when watching heartwarming political ads by their own party and that this motivated them to contribute to their party’s efforts. Kama muta is a positive affective experience, but that does not imply that it is inherently connected to equality or social harmony between groups. Instead, it should strengthen any moral conviction of the group that it is experienced with. However, Although it is not inherently connected to social equality, kama muta may have the potential to “move” the members of different groups that share similar values and beliefs about justice motivating them to join efforts to achieve positive social change.

## Conclusion

The present research extends the role of being moved for collective action, suggesting an application of kama muta theory to intergroup relations that can lead to collective actions aimed to foster equality among groups. We replicate earlier work showing that the impact of collective efficacy on collective action is partly mediated by the emotional experience of the togetherness, which is typically labeled being moved, and what we conceptualize as kama muta. Simply put, this emphasizes the *collective* in collective efficacy.

## Data Availability Statement

The datasets presented in this study can be found in online repositories. The names of the repository/repositories and accession number(s) can be found below: https://osf.io/aqmxh.

## Ethics Statement

The studies involving human participants were reviewed and approved by the internal review board at the Department of Psychology, University of Oslo. The patients/participants provided their written informed consent to participate in this study.

## Author Contributions

DLP conceptualized the research, created the materials, carried out the investigation, and wrote the first draft of the manuscript. DLP and TS conducted the all statistical analyses. TS and JR edited and reviewed the manuscript. All authors planned the research.

## Conflict of Interest

The authors declare that the research was conducted in the absence of any commercial or financial relationships that could be construed as a potential conflict of interest.

## Publisher’s Note

All claims expressed in this article are solely those of the authors and do not necessarily represent those of their affiliated organizations, or those of the publisher, the editors and the reviewers. Any product that may be evaluated in this article, or claim that may be made by its manufacturer, is not guaranteed or endorsed by the publisher.

## Footnotes

1
https://prolific.co/
