# Supplementary material for: Moved by Social Justice: The Role of Kama Muta in Collective Action Toward Racial Equality
Source: Front Psychol. 2022 Mar 1;13:780615. doi: 10.3389/fpsyg.2022.780615 (PMC8921536; doi:10.3389/fpsyg.2022.780615)
Supplement: Supplementary file 1 [file Data_Sheet_1.pdf]

Supplementary Material

1. **Overview Measure**

1.1 Emotions

Instructions:

In the description below, we describe an emotional state for you, keep in mind that not everyone experiences this emotion in the same way. Please read it carefully and think about what it is like for you to feel this emotion.

EMOTION 1

"This emotion gives you a negative feeling that makes you feel like you want to act to change the situation. Perhaps you have felt this emotion after being blocked from pursuing a goal and/or after perceiving unfairness. When the emotion is mild, you may feel aroused and irritated. When it is intense, you may notice a reddening in your face, an increase in your heart rate, muscle tension, more intense breathing, your jaws clenching, or that you furrow your brow. You would probably call this being angry, furious or indignant."

When thinking about the (target),

- How often did you feel this emotion toward the (Target) during the past year? (1)
  - Never (1); Rarely (2); Sometimes (3); Often (4); Always (5)
- During the past year, how strongly did you typically experience this emotion toward the (Target) ?

  - Not at ll (1); Slightly strong (2); Somewhat strong (3); Moderately strong (4); Very strong (5)
- During the last year, how present was this emotion in your life, toward the (Target)?
  - Not at ll (1); Slightly present (2); Somewhat present (3); Moderately present (4); Very present (5)
- How easily do situations from the last year come to your mind where you felt this emotion toward the (target) ?
  - Not at ll (1); Slightly easy (2); Somewhat easy (3); Moderately easy (4); Very easy (5)

EMOTION 2

"This emotion gives you a positive feeling, and it makes you feel connected to others. Perhaps you have felt this emotion after experiencing a sudden sense of closeness to somebody else or an incredibly strong bond with another person or a group of people. When this emotion is mild, it feels a little warm and fuzzy. When it is more intense, you may notice warmth or a stirring in the centre of the chest; your eyes may tear up, or you actually weep. Some people experience chills or goosebumps. You would probably call this as being touched or moved, and you would perhaps call the situation heart-warming."

When thinking about the (target),

- How often did you feel this emotion toward the (Target) during the past year? (1)
  - Never (1); Rarely (2); Sometimes (3); Often (4); Always (5)
- During the past year, how strongly did you typically experience this emotion toward the (Target) ?
  - Not at ll (1); Slightly strong (2); Somewhat strong (3); Moderately strong (4); Very strong (5)

- During the last year, how present was this emotion in your life, toward the (Target)?
    - Not at ll (1); Slightly present (2); Somewhat present (3); Moderately present (4); Very present (5)
- How easily do situations from the last year come to your mind where you felt this emotion toward the (target)?
    - Not at ll (1); Slightly easy (2); Somewhat easy (3); Moderately easy (4); Very easy (5)

EMOTION 3

“This emotion gives you a negative feeling that makes you feel «down». Perhaps you have felt this emotion after a loss, disappointment or misfortune suffered by yourself or others. When it is mild, you could notice that you sigh and slump. When it is intense, you may experience a drop in your mouth corners, a lump in your throat and tears. You would probably call this being depressed, sad or unhappy.”

When thinking about the (target),

- How often did you feel this emotion toward the (Target) during the past year? (1)
    - Never (1); Rarely (2); Sometimes (3); Often (4); Always (5)
- During the past year, how strongly did you typically experience this emotion toward the (Target) ?
    - Not at ll (1); Slightly strong (2); Somewhat strong (3); Moderately strong (4); Very strong (5)
- During the last year, how present was this emotion in your life, toward the (Target)?
    - Not at ll (1); Slightly present (2); Somewhat present (3); Moderately present (4); Very present (5)

- How easily do situations from the last year come to your mind where you felt this emotion toward the (target) ?
    - Not at ll (1); Slightly easy (2); Somewhat easy (3); Moderately easy (4); Very easy (5)

1.2 Collective action

Please indicate your level of agreement / disagreement with the following statements:

- I would participate in a demonstration against racism
- I would participate in a protest for racial equality
- I would volunteer in an organization or association to fight racism
- I would convince others to engage in racial equality discussions
- I would sign a petition to stop racism
- I would donate to a charity which supports Black people
- I would join an online campaign to post or share information supporting racial equality in social networks
    - Strongly disagree (1) to strongly agree (7)

1.3 Attitudes

Please use this scale to indicate how cold (do not feel favourable = 0 in thenscale), neutral (50 in the scale) or warm (feel favourable = 100 on the scale) towards the following groups:

- Black people
- White people
- US Americans
- The system of racial inequalities
- The Black Lives Matter Movement

1.4 Contact

- On average, how frequently do you have POSITIVE/GOOD contact with Black people?
- On average, how frequently do you have POSITIVE/GOOD contact with White people?
  - Never (1) to Always (7)

1.5 Identity

Please indicate your level of agreement with the following statements:

- I identify with Black people
- I identify with White people
- I identify with US Americans
- I identify with The Black Lives Matter movement
- I identify with Racial justice activists
  - Fully disagree (1) to Fully agree (7)

1.6 Support

- Do you support the goal of racial equity?
- Do you support the Black Lives Matter movement?
  - Yes/No

2. Further analysis hypothesis testing for Each group: Collective action regressed on Appraisals

The pre-registration also specified that the model would be run separately for both groups. For this, we also ran the same model for each group separately, without main effects and interaction terms of ethnicity. For White participants, 40.1% of variance in collective action was explained by the model, $F(2, 104) = 34.77$, $p < .001$. For Black participants, 12.4% of the variance was explained, $F(2,105) = 7.40$, $p < .001$. For White participants, collective efficacy, $B = .77$ [.47, 1.09], $\beta = .41$, $p <$

.001 and unfairness, *B* = .75 [.39, 1.10], *β* = .35, *p* < .001 increased collective action intentions. However, for Black participants, collective efficacy increased collective action, *B* = .49 [.21, .76], *β* =.32 ,*p* = .001 but unfairness did not, *B* = .40 [-.02, .82], *β* = .17, *p* = .060.

3. Exploratory Analysis main study

Additional analyses were conducted to explore the association between identity, contact, and attitudes toward different actors in the context of the Black Lives Matter movement and collective action. It is important to mention that these exploratory analyses describe the sample but do not elaborate on emotions and collective action. We ran simple correlations between all variables (*See table 4 in supplementary material)*. In addition, we ran three multiple regression models using the stepwise method. They included: 1) Collective action regressed on attitudes toward US Americans, the BLM movement, the System of racial inequalities, Black people and White people; 2) Collective action regressed on identity with US Americans, the BLM movement, Racial Justice activists, White people and Black people; and 3) Collective action regressed on positive contact with Black people and White people. We carried out these analyses with the total sample and for each group of participants. Results are presented below.

Model 1: Collective action regressed on Attitudes. In the total sample the model explained 42% of the variance of collective action *F*(2,212) = 76.88 , *p* < .001, R^2 = .43. The intercept was *B* = 2.11. Attitudes toward the BLM movement *B* = .03 [.02, .03], β = .49, *p* < .001 and attitudes toward White people *B* = .02 [.01, .03], β = .23, *p* < .001 increased intentions to participate in collective action. No other predictor was significant. After splitting the sample, both attitudes toward the BLM movement and attitudes toward White people were predictors of collective action for both White and Black participants. For White participants, the model explained 63.6% of the variance of collective action *F*(2,104) = 90.93, *p* < .001, R^2 = .63. Attitudes toward the BLM movement *B* = .04 [.03, .04], β

= .66, $p < .001$ and toward White people $B = .02$ [.01, .03], β = .21, $p = .004$ increased collective action. For Black participants the model explained only 25.8% of the variance of collective action $F(2,105) = 18.22$, $p < .001$, $R^2 = .26$. Attitudes toward the BLM movement $B = .02$ [.01, .03], β = .30, $p = .003$ and attitudes towards White people $B = .02$ [.01, .03], β = .28, $p = .006$ increased collective action.

Model 2: Collective action regressed on Identity. In the total sample, the model explained 54% of the variance of collective action $F(3,211) = 82.45$, $p < .001$, $R^2 =.54$. The intercept was $B =$ 2.25. Identification with racial justice activists, $B = .58$ [-.05, .15], β = .65, $p <.001$, and identification with the BLM movement $B = .16$ [.03, .28], β = .19, $p =.013$. Increased intentions to participate in collective action. However, identification with Black people, $B = -.12$ [-.20, -.04], β = .65, $p =.005$ decreased it. Other predictors were not significant.

After splitting the sample based on ethnicity, the model explained 58.4% of the variance of collective action for White participants $F(2,104) = 73.07$, $p <.001$ and 61.4 % for Black participants $F(2,105) = 83.48$, $p <.001$. Identification with racial justice activists increased collective action in White participants $B = .38$ [.19, .59], β = .41, $p < .001$ and Black participants, $B = .63$ [.05, .45], β = .69, $p < .001$. In addition, identity with the BLM movement increased collective active intentions only for White participants $B = .39$ [.19, .59], β = .40, $p < .001$. Identification with Black people increased intentions to participate in collective action only for Black participants $B = .25$ [.05, .45], β = .17, $p = .013$. Other variables were not predictors in any of both groups.

Model 3: Collective action regressed on Contact. After splitting the sample, the model explained 10.6% of the variance of collective action $F(1,105) = 12.435$, $p < .001$, for White participants. For Black participants the model explained 16.5% of the variance of collective action $F(1,106) = 20.901$, $p <.001$. Positive good contact with Black people was positively associated with

intentions to participate in collective action for White participants $B = .42$ [.18, .65], $\beta = .33$, $p = .001$ and for Black participants, $B = .68$ [.38, .97], $\beta = .41$, $p < .001$. Contact with White people was not associated to intention to participate in collective action for neither Black nor White participants.

Discussion

The exploratory analysis suggests that attitudes, identity and contact were predictors of collective action intention toward racial justice. The strongest effect of attitudes was attitudes toward the BLM for both groups. The warmer the attitudes toward the movement, the higher the intentions to participate in collective action, this effect was particularly stronger in White participants. Regarding identity, Identification with racial justice activists had the strongest effect for both groups. Also, identification with the BLM movement increased participation in collective action only for White participants. Instead, for Black participants identification with Black people increased participation in collective action. Finally, positive good contact with Black people increased participation in collective action for both groups.

Supplementary Figures and Tables

Supplementary Table 1

Descriptive statistics and correlations for main variables in full sample (N=215), main study

	M	*SD*	*1*	*2*	*3*	*4*	*5*	*6*
1.Collective efficacy	*4.04*	*0.90*	*1*					
2.Unfairness	*4.63*	*0.68*	*.17**	*1*				
3.Anger to system	*3.52*	*1.05*	*.31***	*.43***	*1*			
4. Kama muta to movement	*2.93*	*1.16*	*.28***	*.27***	*.46***	*1*		
5.Sadness to system	*3.52*	*1.11*	*.18***	*2.5***	*.56***	*.35***	*1*	
6. Collective action	*5.24*	*1.52*	*.42***	*.35***	*.66***	*.53***	*.45***	*1*

**p < .05. **p < .01.*

Supplementary Table 2

Correlations variables pilot study

	M	*SD*	*1*	*2*	*3*	*4*	*5*	*6*	*7*	*8*	*9*	*10*	*11*	*12*
1. Anger to system	*3.08*	*1.09*	*1*											
2. Anger to Movement	*2.31*	*1.24*	*.229***	*1*										
3. Anger to Black	*1.87*	*1.03*	*.155*	*.450***	*1*									
4.Anger to White	*2.60*	*1.07*	*.336***	*.177*	*.013*	*1*								
5. Kama muta to system	*1.75*	*1.03*	*.050*	*.258**	*.670***	*-.036*	*1*							
6. Kama muta to movement	*2.48*	*1.21*	*.554***	*.151*	*.246**	*.201*	*.264**	*1*						
7. Kama muta to Black	*2.91*	*1.01*	*.336***	*.528***	*.244**	*.088*	*.300***	*.508***	*1*					
8. Kama muta to White	*2.60*	*1.06*	*-.055*	*.283**	*.246**	*.020*	*.172*	*.127*	*.463***	*1*				
9. Sadness to System	*3.27*	*1.25*	*.624***	*.129*	*-.148*	*.153*	*-.080*	*.285**	*.302***	*-.061*	*1*			
10. Sadness to Movement	*2.31*	*1.09*	*.274**	*.454***	*.255**	*.031*	*.238**	*.170*	*.283**	*.064*	*.414***	*1*		

11. Sadness to Black	*2.32*	*1.07*	*.181*	*.231**	*.273**	*.272**	*.330***	*.212*	*.305***	*.406***	*.376***	*.378***	*1*	
12. Sadness to White	*2.57*	*1.12*	*.296***	*-.046*	*-.104*	*.619***	*-.091*	*.263**	*.051*	*.073*	*.403***	*.041*	*.391***	*1*
13. Collective action	*3.54*	*1.18*	*.643***	*.111*	*.184*	*.184*	*.105*	*.707***	*.289**	*-.058*	*.507***	*.246**	*.176*	*.247**

*p < .05. **p < .01.

Supplementary Table 3

Descriptive statistics and correlations for main study variables and social identification, Black Participants

	M	*SD*	1	2	3	4	5	6	7	8	9	10	11
1. Collective efficacy	4.04	0.90	1										
2. Unfairness	4.63	0.68	-.06	1									
3. Anger to system	3.52	1.05	.12	.27*	1								
4.Kama muta to movement	2.93	1.16	.20*	.28*	.37**	1							
5. Sadness to system	3.52	1.11	-.12	.15	.41**	.17	1						
6. Black identification	4.85	2.01	.15	.35**	.41**	.27*	.13	1					
7. White identification	4.43	1.77	.23*	-.18	-.07	.11	.15	.20*	1				
8. American identification	5.01	1.59	.17	-.07	-.11	-.03	-.28*	.17	.35**	1			
9. BLM Identification	4.69	1.83	.32**	.11	.22*	.60**	.08	.31*	.21*	.15	1		
10. Activist Identification	5.02	1.71	.37**	.27*	.46**	.53**	.10	.45**	.12	.08	.72**	1	
11. Ideology	3.86	2.58	.07	-.26*	-.18	.00	-.14	-.11	.34**	.24*	-.12	-.14	1
12. Collective action	5.24	1.52	.31*	.16	.60**	.51**	.20*	.48**	.11	.10	.59**	.77**	-.56**

*$p < .05$. **$p < .01$.

Supplementary Table 4

Descriptive statistics and correlations for main study variables and social identification, White participants

	M	*SD*	1	2	3	4	5	6	7	8	9	10	11
1. Collective efficacy	4.04	0.90	1										
2. Unfairness	4.63	0.68	.39**	1									
3. Anger to system	3.52	1.05	.50**	.53**	1								
4.Kama muta to movement	2.93	1.16	.36**	.28*	.53**	1							
5. Sadness to system	3.52	1.11	.55**	.34**	.73**	.60**	1						
6. Black identification	4.85	2.01	.32**	.08	.23*	.21*	.28*	1					
7. White identification	4.43	1.77	-.05	-.09	.04	.15	.01	.08	1				
8. American identification	5.01	1.59	.00	-.01	-.07	-.10	-.06	.14	.54**	1			
9. BLM Identification	4.69	1.83	.41**	.35**	.63**	.41**	.58**	.41**	0.63	.034	1		
10. Activist Identification	5.02	1.71	.45**	.35**	.62**	.43**	.63**	.46**	.045	-.031	.80**	1	
11. Ideology	3.86	2.58	-.27*	-.28*	-.50**	-.25*	-.44**	-.03	.25*	.28*	-.53**	-.50**	1
12. Collective action	5.24	1.52	.55**	.51**	.73**	.56**	.71**	.32**	.02	-.02	.72**	.73**	.-56**

*$p < .05$. **$p < .01$.

Supplementary Table 5

Descriptive statistics and correlations for study variables (identity and contact), main study, full sample

	M	*SD*	1	2	3	4	5	6	7	8	9	10	11
1.Collective efficacy	4.04	0.90	1										
2.Unfairness	4.63	0.68	.170*	1									
3.Anger to system	3.52	1.05	.308**	.403**	1								
4.Kama muta to movement	2.93	1.16	.277**	.267**	.460**	1							
5.Sadness to system	3.52	1.11	.176**	2.47**	.560**	.350**	1						
6.Contact black	5.52	1.11	.165*	.134*	.254**	.286**	.177**	1					
7.Contact white	5.15	.989	.159*	.030	-.045	.027	-.045	.369*	1				
8.Black identity	4.85	2.01	.200**	.101	.289**	.276**	.256**	.449**	-.073	1			
9.White identity	4.43	1.77	.089	.-.105	-.082	.019	-.156*	-.064	.437**	-.379**	1		
10.Movement Identity	4.69	1.83	.352**	.210**	.436**	.534**	.296**	.389**	.018	.469**	-.035	1	
11.Activist Identity	5.02	1.71	.403**	.295**	.554**	.501**	.356**	.383**	-.052	.499**	-.065	.775**	1
12. Collective action	5.24	1.52	.422**	.353**	.664**	.527**	.450**	.343**	.009	.256**	.057	.617**	.716**

**p* < .05. ***p* < .01.

Supplementary Table 6

Descriptive statistics and correlations for study variables (attitudes - ideology), main study, full sample

	M	*SD*	*1*	*2*	*3*	*4*	*5*	*6*	*7*	*8*	*9*	*10*	*11*
1.Collective efficacy	4.04	0.90	1										
2.Unfairness	4.63	0.68	.170*	1									
3.Anger to system	3.52	1.05	.308**	.403**	1								
4.Kama muta to movement	2.93	1.16	.277**	.267**	.460**	1							
5.Sadness to system	3.52	1.11	.176**	2.47**	.560**	.350**	1						
6. Attitudes black	66.1	25.3	.147*	-.088	-.079	.081	-.168*	1					
7.Attitudes white	80.4	21.9	.220*	.301**	.407**	.345**	.282**	.360**	1				
8.Attitudes system	14.0	24.1	.066	-.209**	-.168*	.051	-.213**	.300**	-.067	1			
9. Attitudes BLM	70.7	28.8	.389**	.363**	.505**	.569**	.356**	.096	.552**	-.126	1		
10. Ideology	3.86	2.58	-.083	-.269**	-.318**	-.085	-.273**	.281**	-.104	.295**	-.349**	1	
11. Collective action	5.24	1.52	.422**	.353**	.664**	.527**	.450**	.106	.504**	-.121	.618**	-.372**	1

*p < .05. **p < .0

Supplementary Table 7

Modification indices, model with all parameters equal for both groups, modeling in main study

	White Participants		Black participants	
	M.I	Par change	M.I	Par change
Unfairness <-> Collective efficacy	3.89	.10	5.16	-.14
Sad system <- Collective efficacy	5.40	.19	9.71	-.34
Collective action <- Sad system	2.04	.14		
Sad system <- kama muta movement			3.28	-.16
